# Mechanical ventilation induces brainstem inflammation in preterm fetal sheep

**DOI:** 10.3389/fped.2023.1225294

**Published:** 2023-10-23

**Authors:** Kayla Vidinopoulos, Zahrah Azman, Ainsley Somers, Valerie A. Zahra, Alison Thiel, Hui Lu, Yen Pham, Nhi Thao Tran, Beth J. Allison, Eric Herlenius, Stuart Hooper, Robert Galinsky, Graeme R. Polglase

**Affiliations:** ^1^ The Ritchie Centre, Hudson Institute of Medical Research, Melbourne, VIC, Australia; ^2^ Department of Obstetrics and Gynaecology, Monash University, Melbourne, VIC, Australia; ^3^ Department of Women’s and Children’s Health, Astrid Lindgren Children’s Hospital, Karolinska Institutet, Stockholm, Sweden

**Keywords:** brainstem, mechanical ventilation, ventilation-induced brain injury, inflammation, preterm birth

## Abstract

**Background:**

Preterm infants have immature respiratory drive and often require prolonged periods of mechanical ventilation. Prolonged mechanical ventilation induces systemic inflammation resulting in ventilation-induced brain injury, however its effect on brainstem respiratory centers is unknown. We aimed to determine the effects of 24 h of mechanical ventilation on inflammation and injury in brainstem respiratory centres of preterm fetal sheep.

**Methods:**

Preterm fetal sheep at 110 ± 1 days (d) gestation were instrumented to provide mechanical ventilation *in utero*. At 112** ±** 1 d gestation, fetuses received either mechanical ventilation (VENT; *n* = 7; 3 ml/kg) for 24 h, or no ventilation (CONT; *n* = 6). At post-mortem, fetal brainstems were collected for assessment of mRNA and histological markers of inflammation and injury.

**Results:**

*In utero* ventilation (IUV) did not alter any blood-gas parameters. IUV significantly increased systemic IL-6 and IL-8 concentrations over the 24 h period compared to CONT. The number of ameboid microglia within the nucleus tractus solitarius and the raphe nucleus increased in VENT fetuses (*p* < 0.05 for both vs. control). The % area fraction of GFAP + staining was not significantly higher within the preBötzinger complex (*p* = 0.067) and retrotrapezoid nucleus and parafacial respiratory group (*p* = 0.057) in VENT fetuses compared to CONT. Numbers of caspase-3 and TUNEL-positive cells were similar between groups. Gene expression (mRNA) levels of inflammation, injury, cell death and prostaglandin synthesis within the brainstem were similar between groups.

**Conclusion:**

Mechanical ventilation induces a systemic inflammatory response with only moderate inflammatory effects within the brainstem respiratory centres of preterm fetal sheep.

## Introduction

Preterm birth (<37 weeks’ gestation) is a major contributor to neonatal mortality globally (1, 2) and is also a risk factor for lifelong morbidity including motor deficits such as cerebral palsy and learning deficits (3). Preterm newborns have an immature respiratory system and as such, are often unable to breathe on their own at birth. Consequently, the majority (60%–95%) of extremely and very preterm infants require respiratory support at birth (4), with a median duration of 21.4 days per infant (5, 6). Furthermore, 30% of intubated and ventilated infants fail to resume unassisted breathing upon extubation because of poor respiratory drive (7). It is therefore critical to understand how prolonged mechanical ventilation affects respiratory drive in extremely preterm infants to develop strategies that enhance postnatal breathing.

The brainstem is responsible for controlling vital autonomic functions, including breathing. The brainstem contains multiple bundles of neurons responsible for different aspects of respiratory function, termed respiratory centres (8–10). The brainstem respiratory centres control and generate respiratory rhythm and depth, expiratory and inspiratory timing, opening, and closing of the larynx, and innervate the diaphragm and the tongue (8–10). Recent studies have demonstrated that inflammation within brainstem respiratory centres is associated with inhibition of breathing (11, 12). Exposure of rat pups (10–12 days postnatal age) to intrapulmonary lipopolysaccharide (LPS) upregulated cytokine expression, particularly IL-1β, within the brainstem (13). This brainstem inflammatory response was shown to be at least partially mediated by the vagus nerve and was associated with attenuated ventilatory responses to hypoxia (13). Furthermore, progressive systemic inflammation induced by increasing doses of intravenous LPS in late gestation fetal sheep resulted in inhibition of fetal breathing movements *in utero,* astrocyte loss in the retrotrapezoid nucleus and parafacial respiratory group (RTN/pFRG) and activation of microglia in the RTN/pFRG, the preBötzinger complex (pre-Bötc), nucleus tractus solitarius (NTS) and the raphe nucleus (RN) (14, 15). Increased circulating prostaglandin concentrations have been shown to independently modulate breathing rhythm (16). Upregulation of prostaglandin E synthase (PGE_2_) within the brainstem is also associated with blunting of chemosensitivity and reduced fetal breathing movements *in utero* (12, 17). Taken together, these data suggests that systemic inflammation can directly impact brainstem cardiorespiratory centres and inhibit breathing. While preterm human and animal studies have demonstrated a strong association between mechanical ventilation, systemic inflammation and subsequent brain injury and neurodevelopmental impairments (18–23), the effect of prolonged mechanical ventilation on brainstem inflammation and injury is unknown.

We have previously established a model of *in utero* ventilation (IUV) in preterm fetal sheep (24, 25). Previous IUV studies have limited their ventilation durations to 12 h. In this study we have expanded the timing of ventilation to understand the relationship between prolonged ventilation, systemic inflammation, and localised brainstem inflammation and injury. Using this model, we can determine the impact of mechanical ventilation in the absence of potential confounders of brainstem injury associated with delivery and intensive care of preterm lambs *ex utero,* which at this gestational age have lungs equivalent to a human at 22–24 weeks of gestation (26). These include oxygen supplementation, corticosteroids, nutrition, and anaesthetics. In this study, we aimed to determine the effect of 24 h of mechanical ventilation on inflammation and injury in key brainstem respiratory centres. We hypothesised that 24 h of IUV would increase systemic pro-inflammatory cytokines and result in histological evidence of inflammation and injury in brainstem respiratory centres of preterm fetal sheep at a time when brain development is comparable to a very to moderately preterm human infant (27, 28).

## Materials and methods

### Ethics approval

All procedures were approved by the Hudson Institute of Medical Research Animal Ethics committee [approval number MMCA (Monash Medical Centre Animal Ethics Committee) 2020/15]. All methods were conducted in accordance with the National Health and Medical Research Council Code of Practice for the Care and Use of Animals for Scientific Purposes (Eighth Edition).

### Fetal surgery

Thirteen pregnant Mixed Breed ewes bearing singleton or twin fetuses underwent aseptic surgery at 110 ± 1-day gestational age (term = 147 days). Fetuses were then randomised to one of two groups:

(1) Un-ventilated controls (CONT; *n* = 6)
(2) *In utero* ventilation (VENT; *n* = 7)

Food but not water was withheld approximately 16–18 h before surgery. Ewes were anaesthetised by i.v injection of sodium thiopentone (20 ml) and maintained using 2%–3% isoflurane in oxygen (Bomac Animal Health, Hornsby, NSW, Australia) via a positive pressure ventilator (EV500 Anaesthesia Ventilator, ULCO Medical Engineering, NSW, Australia). Ewes received prophylactic antibiotics (Ampicillin, 1 g i.v; Austrapen, Lennon Healthcare, St Leonards, NSW, Australia, and 500 mg engemycin i.v; Coopers Animal Health, VIC, Australia) before surgery. Levels of isoflurane, heart rate and respiratory rate were continuously monitored throughout surgery by trained anaesthetic staff.

### Fetal instrumentation

**Figure 1:**
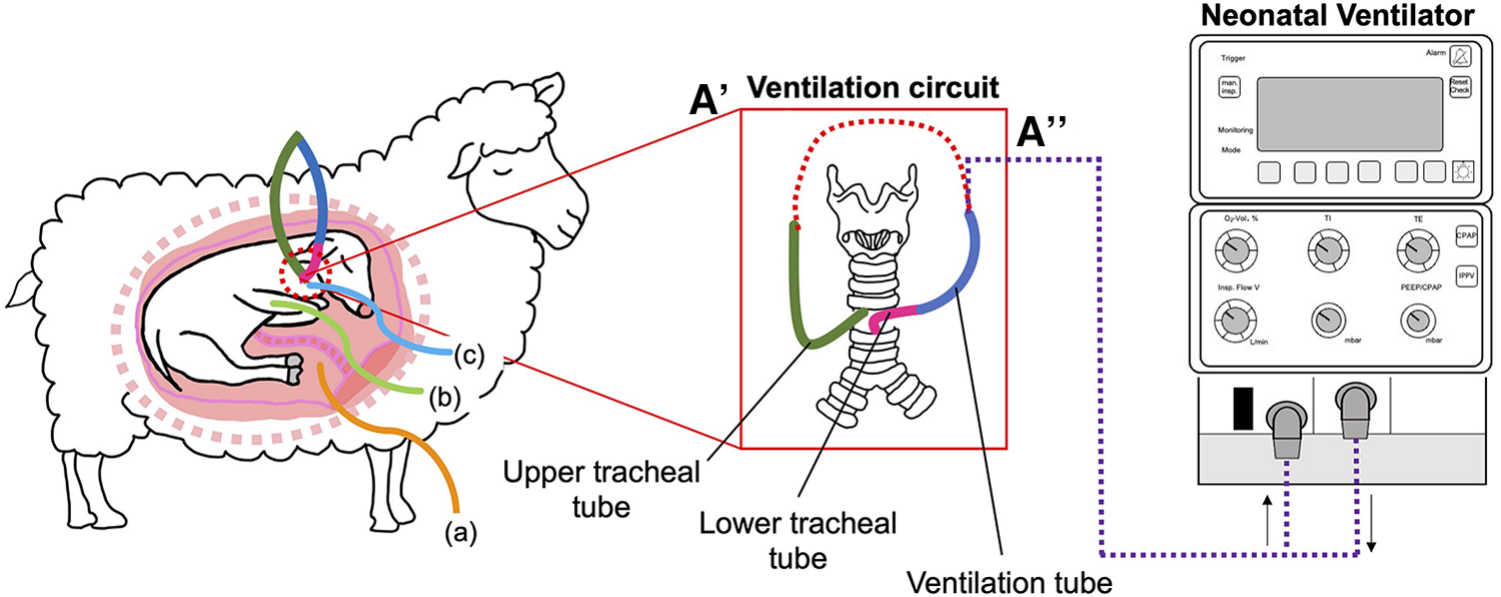
Schematic outlining the *in-utero* ventilation (IUV) experimental design. At 109 d gestation, fetuses were instrumented with a mechanical ventilation circuit consisting of a lower (pink) tracheal tube, connected to a large bore (blue) ventilation tube. The upper (green) tracheal tube was connected to the mechanical ventilation tube (**A**) to create an extension of the trachea, allowing for normal flow of lung liquid prior to initiation of IUV. At the initiation of the experiment, the ventilation circuit was cut, and the tube connected to the ventilator (A’’). Fetuses were instrumented with catheters inserted into the (**A**) amniotic sac, (**B**) brachial artery and (**C**) the jugular vein.

A midline maternal laparotomy was performed to expose the fetus. The fetal head and the left forelimb were exteriorised for instrumentation. Instrumentation procedures and details of catheters have been previously described (24). Briefly, fetuses randomised to the VENT group, underwent a tracheostomy procedure, and were instrumented with a ventilation circuit (Figure 1). Fetuses randomised to CONT also underwent a tracheostomy procedure, but a single non-occlusive tracheal catheter (ID 8.6 mm, OD 3.46 mm) was inserted. All fetal catheters were exteriorised via the right maternal flank. Postoperative analgesia was maintained for 3 days via a transdermal fentanyl patch on the left hind leg of the ewe (75 µg/h; Jansen Cilag, North Ryde, NSW, Australia). Antibiotics were administered i.v to the ewe (ampicillin, 800 mg and engemycin, 500 mg) and the fetus (ampicillin, 200 mg) for 3 consecutive days following surgery. Three days of post-operative recovery were allowed prior to commencing the experiment.

### Experimental protocol

At 112 ± 1 d gestation, the tracheal loop of VENT animals was cut, and lung liquid passively drained prior to initiation of ventilation for 24 h. Lung liquid volume was measured, recorded, and discarded. The tracheal tube was connected to a neonatal ventilator (Drager 8,000+, Lübeck, Germany; Figure 1) and ventilation was initiated in pressure support mode with a peak inflation pressure (PIP) set to a maximum of 45 cmH_2_O and a positive end-expiratory pressure of 5 cmH_2_O to target a tidal volume (V_T_) of 3 ml/kg. An inspiratory flow of 10 L/min, respiratory rate of 60 breaths/min and fraction of inspired oxygen (FiO_2_) of 0.21 were used. Fetuses were ventilated with non-humidified gas.

**Figure 2:**
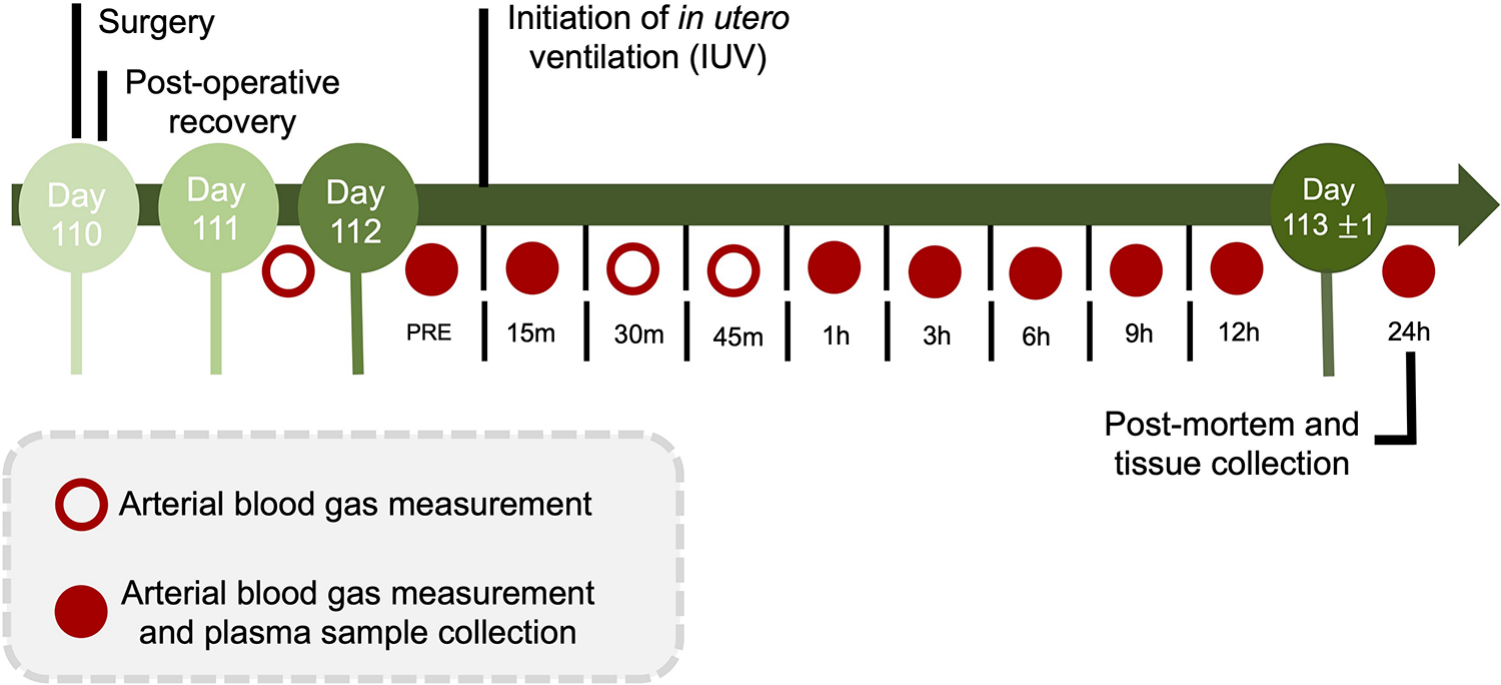
Experimental design and timeline. During the 24 h protocol, arterial blood gas measurements and plasma sample collection occurred at baseline (PRE), 15 min, and 1, 3, 6, 9, 12 and 24 h (red circles). Additional arterial blood gas measurements were made on day 111 and day 112 at 30 and 45-minutes after starting *in utero* ventilation. Following the 24 h IUV or sham IUV period, ewes and fetuses were euthanised.

Fetal arterial blood and plasma samples were collected at baseline (prior to experimentation), at the onset of ventilation (or equivalent for CONT groups), at 15, 30, and 45 min, and at 1, 3, 6, 9, 12 and 24 h after starting ventilation for analysis of blood gases, glucose and lactate concentrations (ABL90 Flex Plus analyser, Radiometer, Brønshøj, Denmark) and for plasma collection for cytokine analysis (Figure 2).

Following 24 h of ventilation (or equivalent for CONT groups), ewes and fetuses were humanely killed via an overdose of sodium pentobarbitone (100 mg/kg i.v., Virbac, NSW, Australia).

### Tissue collection

**Figure 3:**
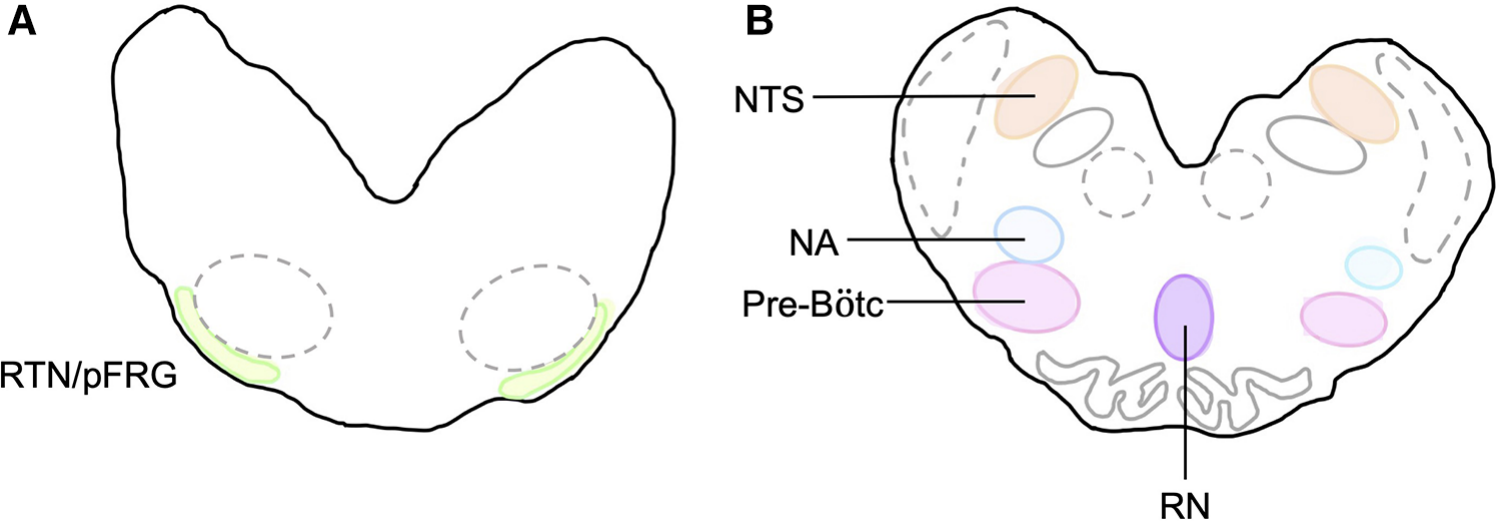
Brainstem respiratory centres of interest. Schematic diagram of brainstem respiratory centres analysed and representation of location in the medulla. (**A**) RTN/pFRG and (**B**) Pre-Bötc**,** NA, NTS, and RN.

At post-mortem, cerebral spinal fluid (CSF) was collected from the 4th ventricle before the brain was removed. The brainstem was isolated at the levels of the peduncles and thalamus, bisected in the sagittal plane before the left side was dissected into the pons and medulla with portions of each frozen in liquid nitrogen. The right brainstem was immersion-fixed in 10% phosphate buffered formalin for 7 days at 4°C before it was embedded and sectioned as previously described (14). Brainstem respiratory centres of interest included the retrotrapezoid nucleus and parafacial respiratory group (RTN/pFRG), the preBötzinger complex (pre-BÖTC), the nucleus tractus solitarius (NTS), the raphe nucleus (RN) and the nucleus ambiguus (NA) (Figure 3). The RTN/pFRG is involved with expiration and central chemosensitivity including CO_2_ and pH. The NTS is involved in peripheral chemosensitivity (O_2_, CO_2_, pH). The NA is involved in maintaining upper airway patency, the RN is responsible for central chemosensitivity and the pre-BÖTC for inspiratory generation and drive.

### Fetal cytokine measurements

Arterial blood samples were analysed at timepoints pre-ventilation (CONT equivalent), 1, 3, 6, 9, 12 and 24 h for assessment of plasma proteins IL-6 and IL-8. Plasma proteins IL-6 and IL-8 were quantified by a sandwich enzyme-linked immunosorbent assay (ELISA) kit as described previously (29). Details of the primary, secondary, and tertiary detecting antibodies can be found in Table 1. In brief, flat-bottom 96-well plates (Nunc Maxisorp™) were coated with mouse-anti-sheep IL-6 and mouse-anti-sheep IL-8 antibodies and incubated overnight at 4°C. Plasma samples were diluted with diluting buffer and incubated in duplicates in the 96-well plates for 1 h at room temperature. The next day, the plates were washed and coated with rabbit-anti-sheep IL-6 or rabbit-anti-sheep IL-8 for 1 h at room temperature. Plates were then washed and incubated with horse-radish peroxidase (HRP)-conjugated swine-anti-rabbit Ig for 1 h at room temperature. Plates were washed, plates were developed with 3.3′, 5.5′-tetramethylbenzidine (TMB chromogen solution; Invitrogen, CA, USA) for 20 min in a dark room at room temperature. Reactions were stopped with 0.5M H_2_SO_4_. Plates were quantified on a plate reader at 450 nm (SpectraMax i3 Multi-Mode Platform, Molecular devices, San Jose, CA, USA). Each group has a sample number of 5 per group, due to inability collect plasma samples from one CONT and two VENT fetal sheep during the experimental timeline.

**Table 1:** Reagents used in enzyme-linked immunosorbent assays for detection of plasma IL-6 and IL-8.

Target	Type	Reagent details	Dilution	Source
IL-6	Cytokine standard	Recombinant ovine (ROV) IL-6	5 µg/ml, at 1:50	Kingfisher Biotech^a^ RP0367V-005
	Primary/coating Ab	Mouse anti-sheep IL-6 monoclonal Ab IgG	1:200	Bio-Rad^b^ MCA1659
	Secondary/capturing Ab	Rabbit anti-sheep IL-6 polyclonal Ab	1:500	Bio-Rad^b^ AHP424
	Tertiary Ab	Swine anti-rabbit Ig-HRP conjugated	1:2,000	DAKO^c^ P0217
IL-8	Cytokine standard	ROV IL-8	N/A	Kingfisher Biotech^a^ RP0488V-005
	Primary/coating Ab	Mouse anti-ovine IL-8 monoclonal Ab IgG	1:1,000	Bio-Rad^b^ MCA1660
	Secondary/capturing Ab	Rabbit anti-ovine IL-8 polyclonal Ab	1:4,000	Bio-Rab^b^ AHP425
	Tertiary Ab	Swine anti-rabbit Ig/HRP conjugated polyclonal Ab	1:2,000	DAKO^c^ P0217

^a^ Kingfisher: Kingfisher Biotech, Inc., MN, USA.

^b^ Bio-Rad: Bio-Rad Laboratories, Inc., CA, USA.

^c^ DAKO: Agilent Technologies, Inc., CA, USA.

### Fetal cerebrospinal fluid (CSF) protein analysis

CSF samples collected at post-mortem were assessed for proteins IL-1β, IL-6, IL-8. IL-10, tumor necrosis factor (TNF) and interferon *γ*-produced protein (IP-10) using Milliplex MAP bovine cytokine magnetic bead panel assay kits (cat#: BCYT1-33K; MerckMillipore, Burlington, MA, USA) as previously described (15). In brief, 96 well plates were first washed and coated in sample, assay buffer, serum matrix, and antibody-immobilised beads. The plates were incubated overnight at 4°C. Following overnight incubation, the plates were washed and incubated with the detection antibodies for 1 h. Streptavidin-phycoerythrin was added to the plates for 30 min at room temperature. Sheath fluid was then added to each of the plates. Protein concentrations were quantified using a Bio-Plex MAGPIX® Multiplex reader with xPOTENT® software (Bio-Rad, CA, USA). Internal quality controls were included in each assay kit. Cytokine levels were within detection limit in all samples. Standards were bovine recombinant IL-1 β (range, 12.8–200,000 pg/ml; assay sensitivity, 3.9 pg/ml), IL-6 (range, 7.7–120,000 pg/ml; assay sensitivity, 0.65 pg/ml), IL-8 (range, 0.77–12,000 pg/ml; assay sensitivity, 1.62 pg/ml), IL-10 (range. 1.6–25,000 pg/ml; assay sensitivity, 0.86 pg/ml), TNF (range, 48–750,000 pg/ml; assay sensitivity, 9.34 pg/ml), and IP-10 (range, 0.77–12,000 pg/ml; assay sensitivity, 0.46 pg/ml).

### Gene analysis

The left side of the brainstem was weighed to 100–150 mg, homogenised, and mRNA extracted according to manufacturer's instructions using the RNeasy Midi RNA Extraction Kit (Qiagen, Venlo, Netherlands) and reverse transcribed into single stranded cDNA (Superscript III First-Strand Synthesis, Invitrogen, MA, USA). High throughput real-time quantitative polymerase chain reaction (RT-qPCR) was performed using the microfluidic technology Fluidigm Access Array System (Fluidigm Corporation, CA, USA). Genes of interest included inflammatory cytokines interleukin (IL)-*1A, IL-1B, IL-6, IL-8, IL-10, IL-18,* tumor necrosis factor (*TNF);* markers of inflammation nuclear factor kappa B (*NFκB),* CXC motif chemokine ligand 10 (*CXCL-10),* high mobility group box *1 (HMGB1), FOXP3*, matrix metallopeptidase 9 (*MMP-9)*, myeloperoxidase (*MPO)*, serum amyloid A (*SAA),* toll-like receptor (*TLR)*-4*;* markers of cell death/damage, caspase (*CASP)-1, CASP-3, CASP-8*, heat shock protein-70 (*HSP70*); markers of prostaglandin synthesis prostaglandin E synthase (*PTGES)* and prostaglandin-endoperoxide synthase-2 (*PTGS-2).* Quality control testing was performed for the housekeeping genes *18S, B2M* and *S29* using Sybr chemistry. The expression of genes were normalised to housekeeping genes by first calculating a geometric mean and then by subtracting the Ct value for the geometric mean for all samples of housekeeping genes from the Ct value of the genes of interest (ΔCt). mRNA levels of the genes of interest were normalised using the formula 2^−ΔCt^ and the results expressed as fold-change from control. Details of the primers are presented in Table 2.

**Table 2:** List of taqMan gene array ovine-specific probes for qPCR.

Genes/Probe	Assay ID
IL-1α	Oa04658682_m1
IL-1β	Oa04656322_m1
IL-6	Oa04656315_m1
IL-8	Bt03211906_m1
IL-10	Oa03212724_m1
IL-18	Oa04658606_m1
TNF	Oa04656867_g1
NFκB	Oa04837805_m1
CXCL-10	Oa04655788_g1
HMGB1	Ch04812286_s1
FOXP3	Oa03233950_g1
MMP-9	Oa03215996_g1
MPO	Oa04654413_g1
SAA	Oa04924154_s1
TLR-4	Oa04656419_m1
CASP-1	Oa04775045_m1
CASP-3	Oa04817361_m1
CASP-8	Oa04779925_m1
HSP70	Oa04849683_g1
PTGES	Oa04920211_s1
PTGS-2	Oa04657348_g1
S18	Oa4906333_g1
B2M	Oa04900279_Mh
S29	Ch04807765_gH

### Immunofluorescence

Immunofluorescence was used to double label astrocytes [mouse anti-glial fibrillary acidic protein (GFAP), 1:500, Sigma, cat#. G3893], and microglia [rabbit anti-ionized calcium binding adaptor molecule (Iba-1), 1:500, Wako, 019–19,741]. Brainstem sections were incubated at 60°C for 30 min and then dewaxed in xylene, rehydrated in ethanol, and washed in 0.1 mol/l phosphate buffer saline (PBS; pH 7.4). Heat mediated antigen retrieval was performed in citrate buffer (pH 6) using a microwave for 15 min. 10% normal goat serum (NGS) in 0.1% PBS + Triton X-100 (PBST) was used for non-specific antigen blocking. Sections were incubated in primary antibody and 0.1% PBST and 2% NGS overnight at 4°C. Negative controls omitting the primary antibody were included to confirm the absence of non-specific staining. Sections were incubated in 1:200 goat anti-mouse- Alexa Fluor 594 (Cat#: 115-585-003, Jackson ImmunoResearch, West Grove, PA, USA) and 1:200 goat-anti-rabbit- Alexa Fluor 488 (Cat#: 111-545-144, Jackson ImmunoResearch) in 0.1% PBST and 2% NGS for 2 h at room temperature. 1:1,000 HOECHST 33342 trihydrochloride, trihydrate (Cat#: H3570, Invitrogen, ThermoFisher Scientific) was used for nuclei staining, slides incubated for 5 min. Slides were washed in PBS and coverslipped using DAKO anti-fade mounting medium (Cat#: GM30411-2, Agilent technologies, CA, USA).

### Immunohistochemistry

Brainstem slides were baked at 60°C for 30 min, de-waxed and rehydrated. Antigen retrieval was performed in citrate buffer (pH 6) using a microwave for 15 min. Endogenous peroxide quenching was performed by incubating slides in 0.1% H_2_O_2_ in methanol. 3% NGS in 1 × PBS was used to prevent non-specific binding. Sections were labelled with 1:800 rabbit-anti-Caspase 3 (R&D systems, cat#: AF835) in 3% NGS and incubated overnight at 4°C. Slides were then incubated in biotin conjugated IgG [1:200 goat anti-rabbit (DAKO, Victoria, Australia)] secondary antibody for 3 h at room temperature before being incubated in avidin-biotin complex (Sigma-Aldrich) for 45 min. Sections were reacted with 3,3′-diaminobenzidine tetrahydrochloride (Sigma-Aldrich). Terminal deoxynucleotidyl transferase dUTP nick end labelling (TUNEL) was used to identify cells undergoing *in-situ* apoptosis using the ApopTag® Peroxidase Kit as per manufacturer's instructions (Millipore S7100; CA, USA). Brainstem sections stained with both caspase-3 and ApopTag were then incubated in anti-digoxigenin conjugate for 30 min at room temperature, before being incubated with diaminobenzidine peroxidase substrate. PBS was used to stop the reaction. Slides were then dehydrated, and cover slipped.

### Quantification of histology

Sections were imaged at 20 × magnification using QuPath imaging software [Version 0.2.3 (30)]. Four random non-overlapping fields of view (length: 1,200 µm; width: 1920µm; area: 2.3mm^2^) were taken from each section. All sections were coded and assessed by a single observer blinded to the treatment group (KV). Immunoreactivity (% area fraction of staining) or numbers of positive cells were quantified from each brainstem respiratory centre of interest from 2 sections per subject using ImageJ software (v2.00, LOCI, University of Wisconsin). GFAP^+^ astrocyte staining was expressed as % area fraction staining per field of view (FOV). Numbers of microglia (Iba-1^+^ cells) were quantified according to their morphology, either ramified (small cell body with >1 branching process) (31) or ameboid (large cell bodies, with ≤1 branching process) (32, 33). Caspase-3^+^ cells displaying both immunostaining and cell morphology resembling karyorrhexis (nuclear breakdown) or vacuolisation were counted (34). ApopTag (TUNEL)^+^ cells were quantified as total numbers of immunopositive cells. Data are presented for both the whole medulla and the individual cardiorespiratory centres.

### Statistical analysis

Data were analysed using GraphPad Prism Software (version 9; GraphPad Software, CA, United States). Parametric fetal characteristics, brainstem gene expression and histological data were analysed by an un-paired *t*-test. Non-parametric fetal characteristics, brainstem gene expression and histology were analysed using Mann-Whitney test. To account for baseline variability in both groups, circulating cytokines have been expressed as fold-change from baseline. A two-way analysis of variance (ANOVA) with repeated measures (treatment and time as independent factors) was used to analyse blood biochemistry and circulating plasma cytokines. For blood biochemistry and cytokines, when statistical significance was found between groups or groups and time, *post-hoc* comparisons were made using a Holm-Sidak test. For cytokines, area under the curve (p_AUC_) analysis was used to evaluate differences between groups when statistical significance was found between groups or group and time. Power analysis of areal density of GFAP+ astrocytes suggested that the study had 80% power to detect a 25% increase in areal density of GFAP + astrocytes between groups, with an alpha of 0.05. Statistical significance was accepted when *p* < 0.05. Data are presented as scatter plots with mean ± standard error of the mean (SE).

## Results

### Fetal characteristics

Fetal body weight (kg), sex (%male), rate of twin pregnancies (%), and brain weight (g) were not different between CONT and VENT groups (Table 3). Fetuses within the VENT group received a mean V_T_ of 2.8 ml/kg (range 2.5–3.1 ml/kg).

**Table 3:** Fetal characteristics. Data are mean ± SEM.

	CONT	VENT
Number (*n*)	6	7
Body weight (kg)	2.7 ± 0.4	2.6 ± 0.5
Sex (male:female)	4:2	4:3
Twin (twins:singletons)	5:1	4:3
Brain weight (g)	36.3 ± 2.8	35.8 ± 2.6

### Fetal biochemistry

The partial pressure of arterial carbon dioxide and oxygen (PaCO_2_ and PaO_2_), arterial oxygen saturation (SaO_2_) and the concentrations of arterial glucose and lactate were not different between CONT and VENT groups at any of the timepoints assessed (Table 4).

**Table 4:** Arterial blood pH, gases, glucose, and lactate concentrations. Data are mean ± SEM.

	Baseline	15 min	30 min	45 min	1 h	3 h	6 h	9 h	12 h	24 h
pH										
CONT	7.42 ± 0.01	7.42 ± 0.00	7.41 ± 0.01	7.41 ± 0.00	7.41 ± 0.01	7.41±±0.01	7.41 ± 0.01	7.41 ± 0.01	7.41 ± 0.01	7.42 ± 0.01
VENT	7.40 ± 0.01	7.40 ± 0.00	7.40 ± 0.01	7.40 ± 0.00	7.40 ± 0.00	7.40 ± 0.01	7.40 ± 0.00	7.40 ± 0.00	7.40 ± 0.00	7.41 ± 0.00
PaCO_2_										
CONT	48.0 ± 0.91	48.9 ± 0.82	46.9 ± 1.13	46.1 ± 1.37	49.8 ± 1.17	49.7 ± 1.36	49.3 ± 1.52	49.3 ± 1.26	50.3 ± 1.30	49.5 ± 1.07
VENT	49.2 ± 1.01	49.4 ± 1.2	47.9 ± 1.34	48.5 ± 1.31	49.4 ± 1.39	49.8 ± 1.19	49.8 ± 1.67	50.2 ± 1.22	50.8 ± 1.15	50.2 ± 0.85
PaO_2_										
CONT	22.0 ± 0.63	21.6 ± 0.69	22.6 ± 0.95	22.2 ± 0.95	21.2 ± 0.42	22.8 ± 0.65	22.3 ± 0.42	21.9 ± 0.52	21.9 ± 0.41	21.6 ± 0.82
VENT	21.7 ± 0.54	21.7 ± 0.36	22.0 ± 0.64	22.0 ± 0.68	22 ± 0.56	23.4 ± 0.70	22.9 ± 0.54	22.1 ± 0.55	22.1 ± 0.68	23.1 ± 0.52
SaO_2_										
CONT	69.2 ± 2.09	68.0 ± 2.11	70.2 ± 2.25	69.5 ± 3.01	67.9 ± 1.71	70.1 ± 1.40	69.2 ± 1.26	69.5 ± 2.76	69.6 ± 2.01	67.0 ± 2.28
VENT	66.0 ± 2.18	66.1 ± 1.22	66.7 ± 2.39	66.9 ± 2.14	66.4 ± 1.81	69.2 ± 1.66	68.8 ± 1.05	67.8 ± 2.55	66.2 ± 1.73	69.9 ± 1.43
Glucose										
CONT	0.97 ± 0.15	0.93 ± 0.18	0.87 ± 0.17	0.83 ± 0.15	0.83 ± 0.15	0.77 ± 0.15	0.78 ± 0.13	0.95 ± 0.19	0.95 ± 0.14	0.88 ± 0.12
VENT	1.09 ± 0.12	1.04 ± 0.14	1.00 ± 0.12	0.96 ± 0.13	0.96 ± 0.13	0.96 ± 0.09	0.94 ± 0.10	0.96 ± 0.10	1.00 ± 0.12	0.94 ± 0.10
Lactate										
CONT	1.15 ± 0.11	1.15 ± 0.11	1.05 ± 0.11	1.03 ± 0.11	1.05 ± 0.07	1.00 ± 0.07	0.98 ± 0.07	1.12 ± 0.12	1.05 ± 0.08	1.28 ± 0.11
VENT	1.24 ± 0.09	1.20 ± 0.09	1.11 ± 0.07	1.07 ± 0.07	1.10 ± 0.07	1.11 ± 0.07	1.19 ± 0.10	1.16 ± 0.06	1.11 ± 0.07	1.14 ± 0.09

### Plasma cytokines

**Figure 4:**
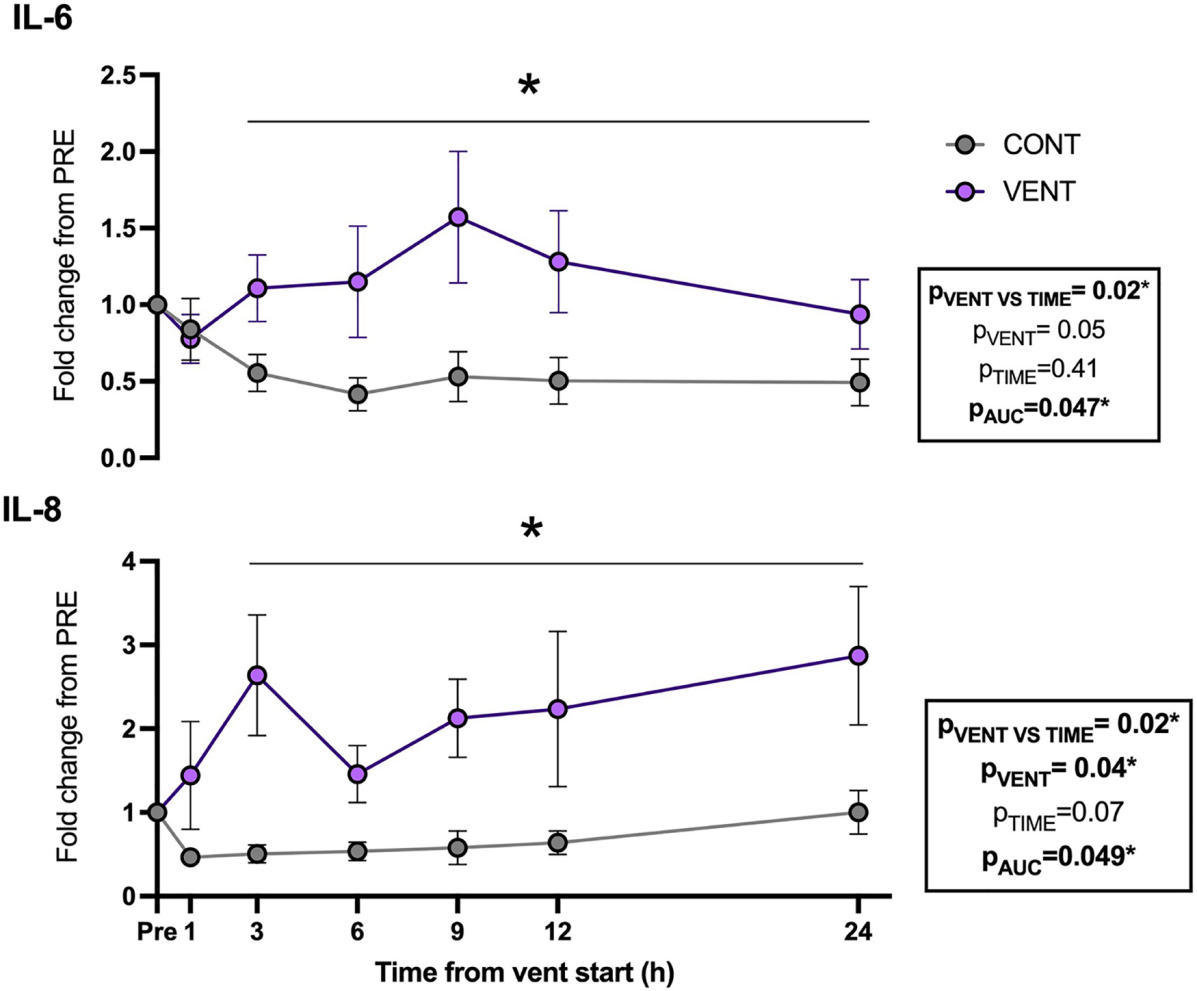
Plasma interleukin (IL)-6- and 8-fold change from baseline (1 h pre-ventilation) for control (CONT; grey circles; *n* = 5) and ventilated (VENT; purple circles; *n* = 5) fetuses over 24 h. Data are mean ± SEM. *p < 0.05.

Plasma IL-6 (p_AUC _= 0.047) and IL-8 (p_VENT _= 0.04) protein concentrations were increased in VENT fetuses compared to CONT fetuses throughout the IUV protocol (Figure 4).

### Cerebrospinal fluid protein

**Figure 5:**
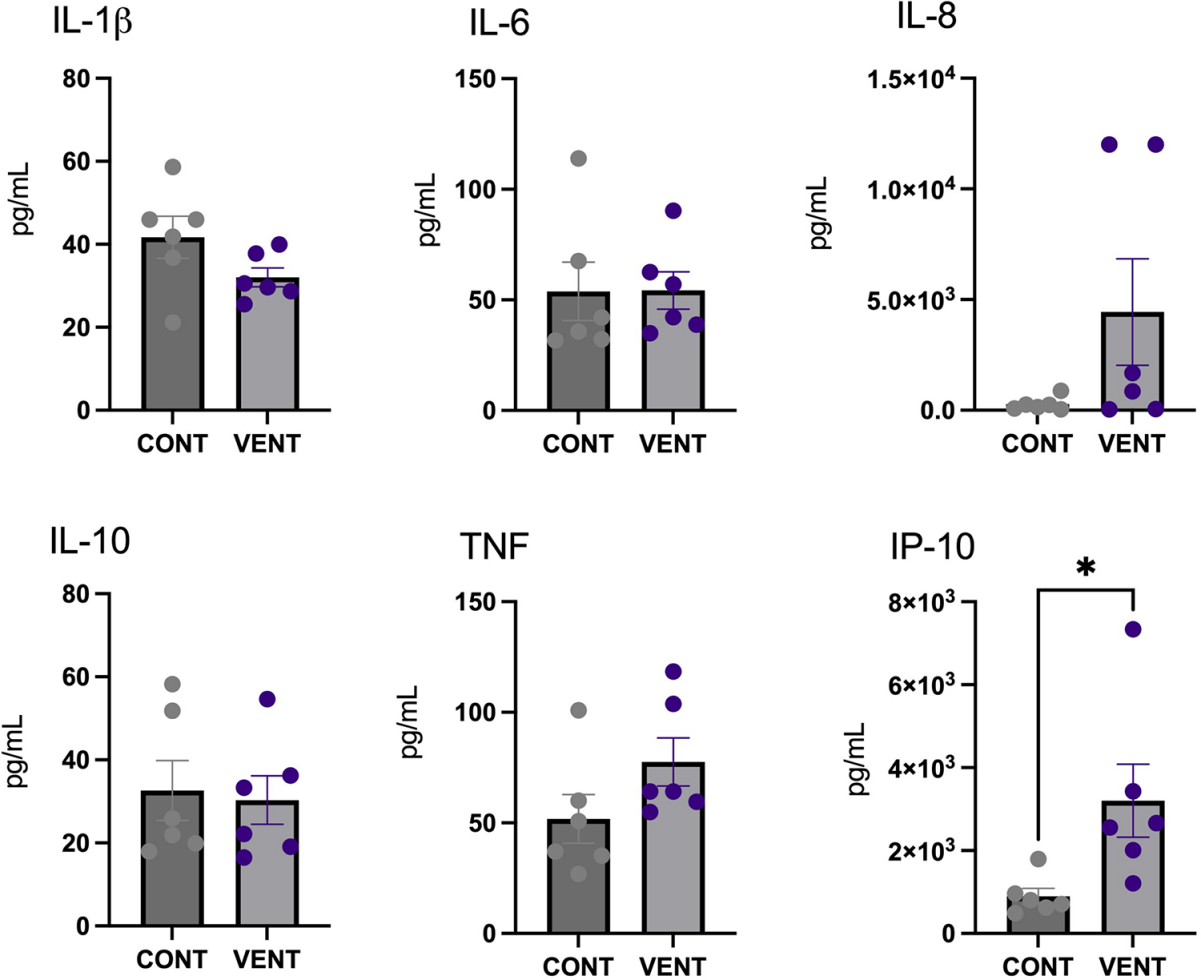
Cerebral spinal fluid (CSF) protein expression of interleukin (IL)-1β, IL-6, IL-8, IL-10, tumor necrosis factor (TNF) and IP-10 in control (CONT; grey circles; *n* = 6) and ventilated (VENT; purple circles; *n* = 6) fetuses. Data are mean ± SEM. *p < 0.05.

In the cerebral spinal fluid, protein concentration of the chemokine IP-10 was increased in VENT exposed fetuses (*p* = 0.0284) compared to CONT. Protein concentrations of IL-1β, IL-6, IL-8, IL-10 and TNF were not different between VENT and CONT groups (Figure 5).

### Gene analysis

**Figure 6:**
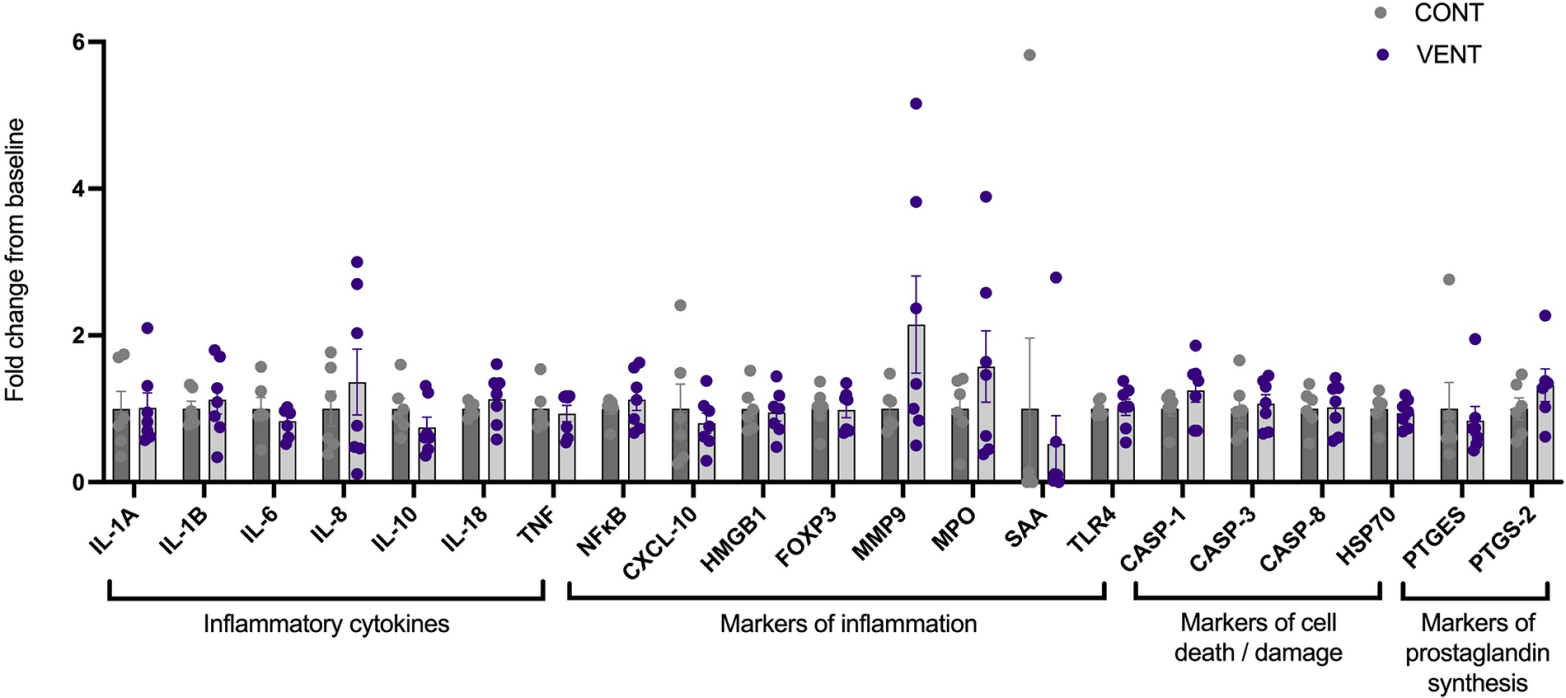
mRNA expression of inflammatory cytokines *IL-1A, IL-1B, IL-6, IL-8, IL-10, IL-18*, *TNF*; markers of inflammation *NF-κB, CXCL-10, HMGB1,* FOXP3, MMP9, MPO, SAA, TLR-4; markers of cell death/damage CASP-1, CASP-3, CASP-8, HSP70; markers of prostaglandin synthesis PTGES, PTGS-2 in control (CONT; grey circles; *n* = 6) and ventilated (VENT; purple circles; *n* = 7) fetuses. Data are mean ± SEM.

In the brainstem, mRNA expression of inflammatory cytokines *IL*-*1A, IL-1B, IL-6, IL-8, IL-10, IL-18, TNF;* markers of inflammation *NFκB*, *CXCL-10, HMGB1, FOXP3*, *MMP-9, MPO*, *SAA, TLR*-4*;* markers of cell death/damage *CASP-1, CASP-3, CASP-8*, *HSP70*; markers of prostaglandin *PTGES* and *PTGS-2* were not different between CONT and VENT groups (Figure 6).

### Histopathology

**Figure 7:**
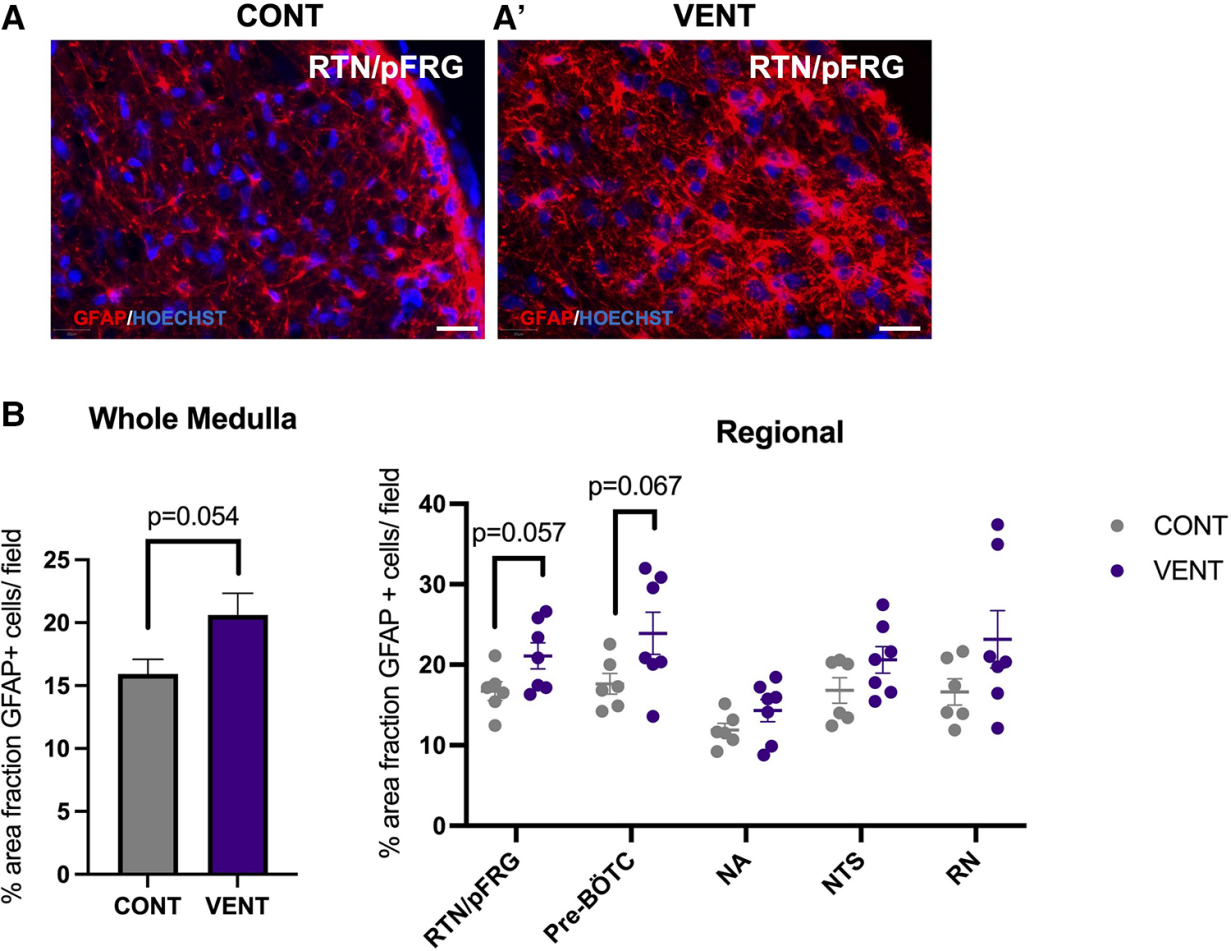
Area fraction of GFAP staining (%) in brainstem respiratory centres. (**A, A’**) Representative photomicrographs of GFAP (red) staining in the retrotrapezoid nucleus and parafacial respiratory group (RTN/pFRG) in CONT and VENT exposed fetuses. Scale = 20 µm (**B**) Percentage area fraction of GFAP + staining in the whole medulla and regional respiratory centres including the retrotrapezoid nucleus and parafacial respiratory group (RTN/pFRG), PreBötzinger complex (Pre- Bötc), nucleus ambiguus (NA), nucleus tractus solitarius (NTS) and raphe nucleus (RN) in control (CONT; grey circles; *n* = 6) and ventilated (VENT; purple circles; *n* = 7) fetuses. Data are mean ± SEM.

**Figure 8:**
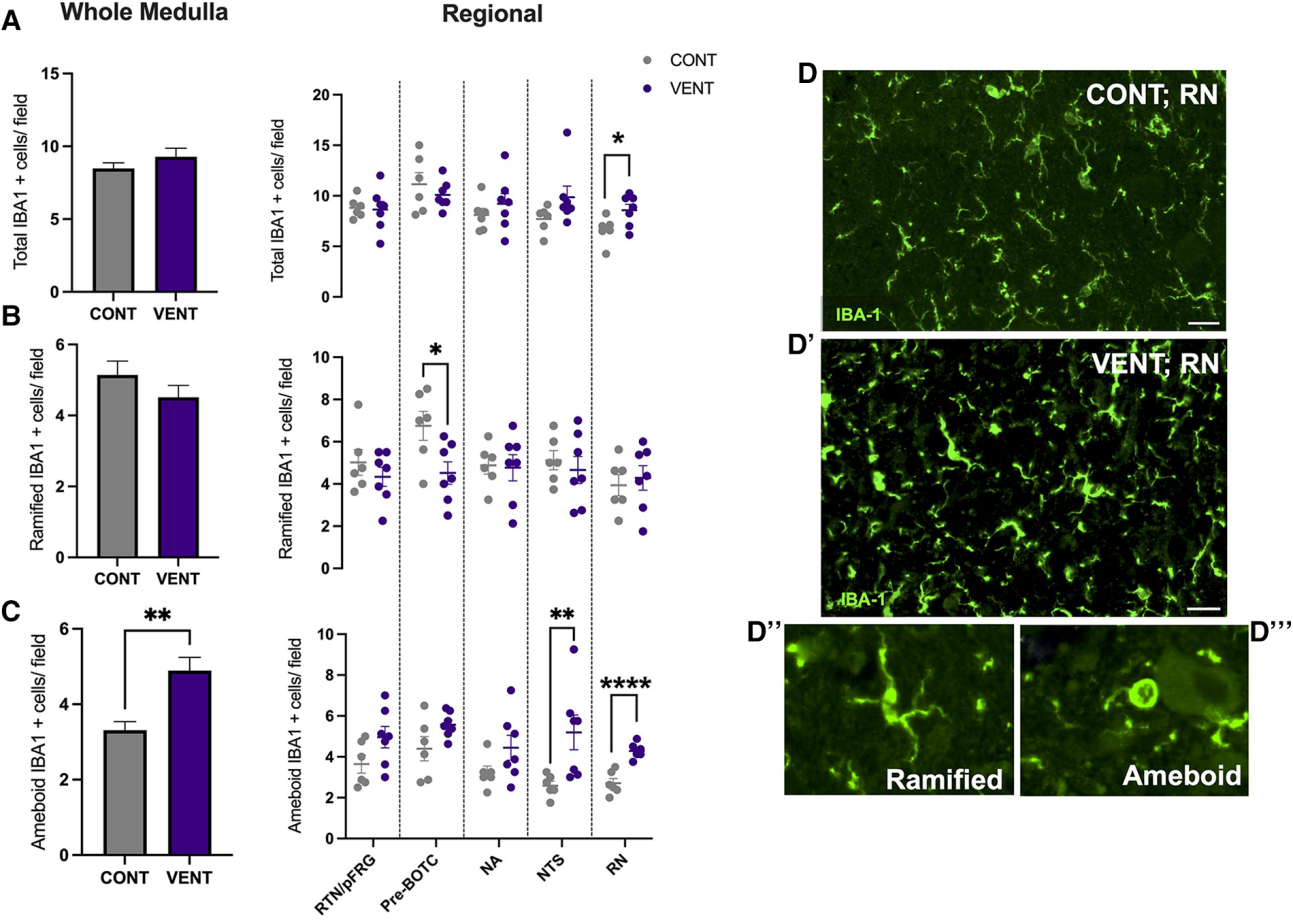
Ionised calcium binding adaptor molecule (IBA-1+) cells in brainstem respiratory centres. (**A**) Total Ionised calcium binding adaptor molecule (IBA-1+), (**B**) Ramified IBA-1 + cells and (**C**) ameboid IBA-1 + cells per field in the whole medulla and regional respiratory centres: retrotrapezoid nucleus and parafacial respiratory group (RTN/pFRG), preBötzinger complex (Pre- Bötc), nucleus ambiguus (NA), nucleus tractus solitarius (NTS) and raphe nucleus (RN) in control (CONT; grey circles; *n* = 6) and ventilated (VENT; purple circles; *n* = 7) fetuses. (**D, D’**) Representative photomicrographs of IBA-1 (green) staining in the RN of CONT and VENT exposed fetuses. Scale = 20 µm. Representative images of ramified (**D’’**) and ameboid (**D’’’**) microglial phenotypes. Data are mean ± SEM. ****p < 0.0001, **p < 0.001, *p < 0.05.

**Figure 9:**
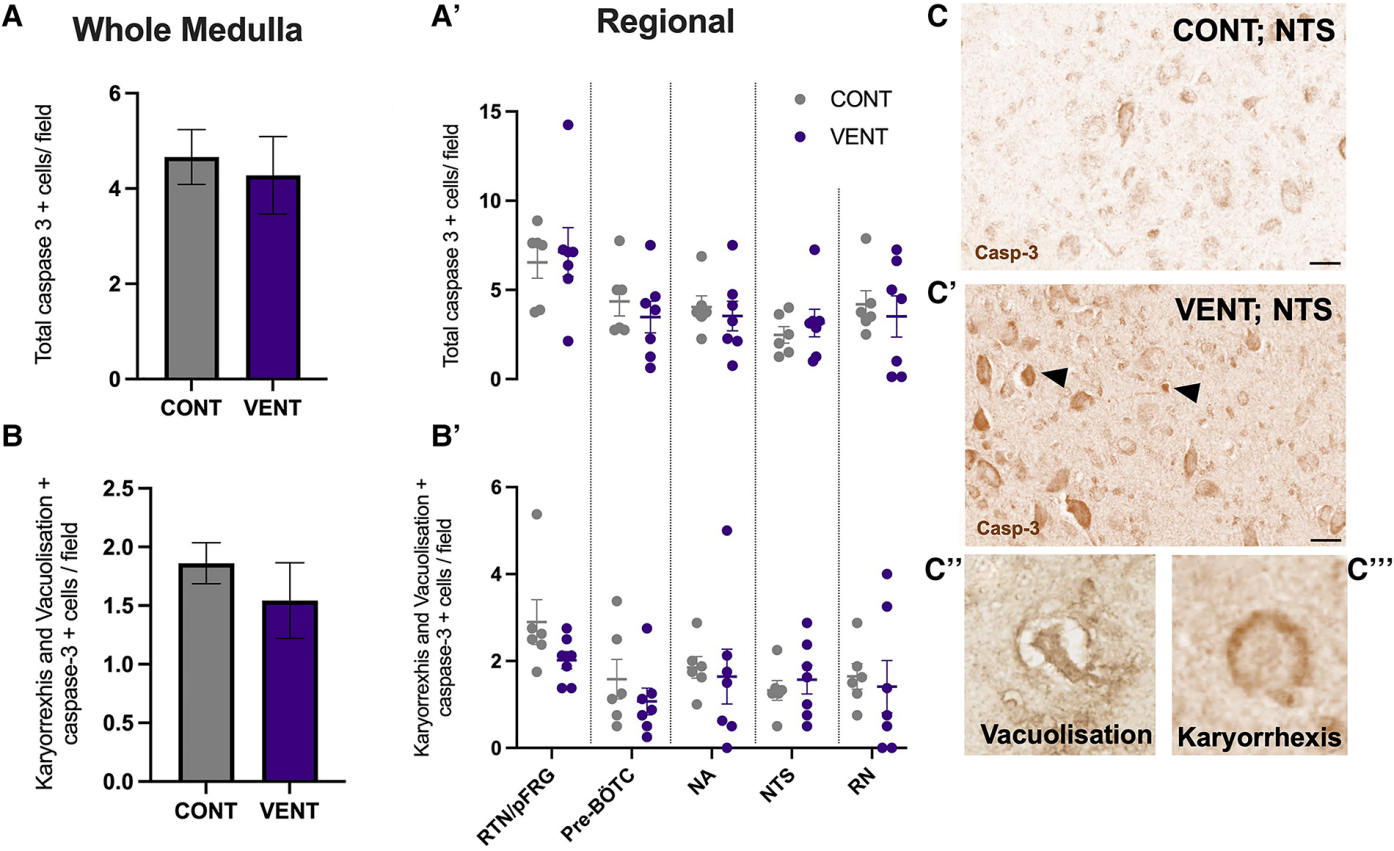
Caspase-3 + cells in brainstem respiratory centres. (**A**) Total caspase-3 positively stained cells per field in the whole medulla and regional respiratory centres (**A’**): retrotrapezoid nucleus and parafacial respiratory group (RTN/pFRG), preBötzinger complex (Pre- Bötc), nucleus ambiguus (NA), nucleus tractus solitarius (NTS) and raphe nucleus (RN). (**B**) Caspase-3 positive cells with morphological features of karyorrhexis or vacuolisation per field in the whole medulla, and regional respiratory centres (**B’**): retrotrapezoid nucleus and parafacial respiratory group (RTN/pFRG), preBötzinger complex (Pre- Bötc), nucleus ambiguus (NA), nucleus tractus solitarius (NTS) and raphe nucleus (RN) in control (CONT; grey circles; *n* = 6) and ventilated (VENT; purple circles; *n* = 7) fetuses. (**C,C’**) Representative photomicrographs of caspase-3 + staining in the NTS of CONT and VENT exposed fetuses. Black arrows point to cells with positive staining. Scale = 20 µm. Representative images of vacuolisation (**C’’**) and karyorrhexis (**C’’’**). Data are mean ± SEM.

**Figure 10:**
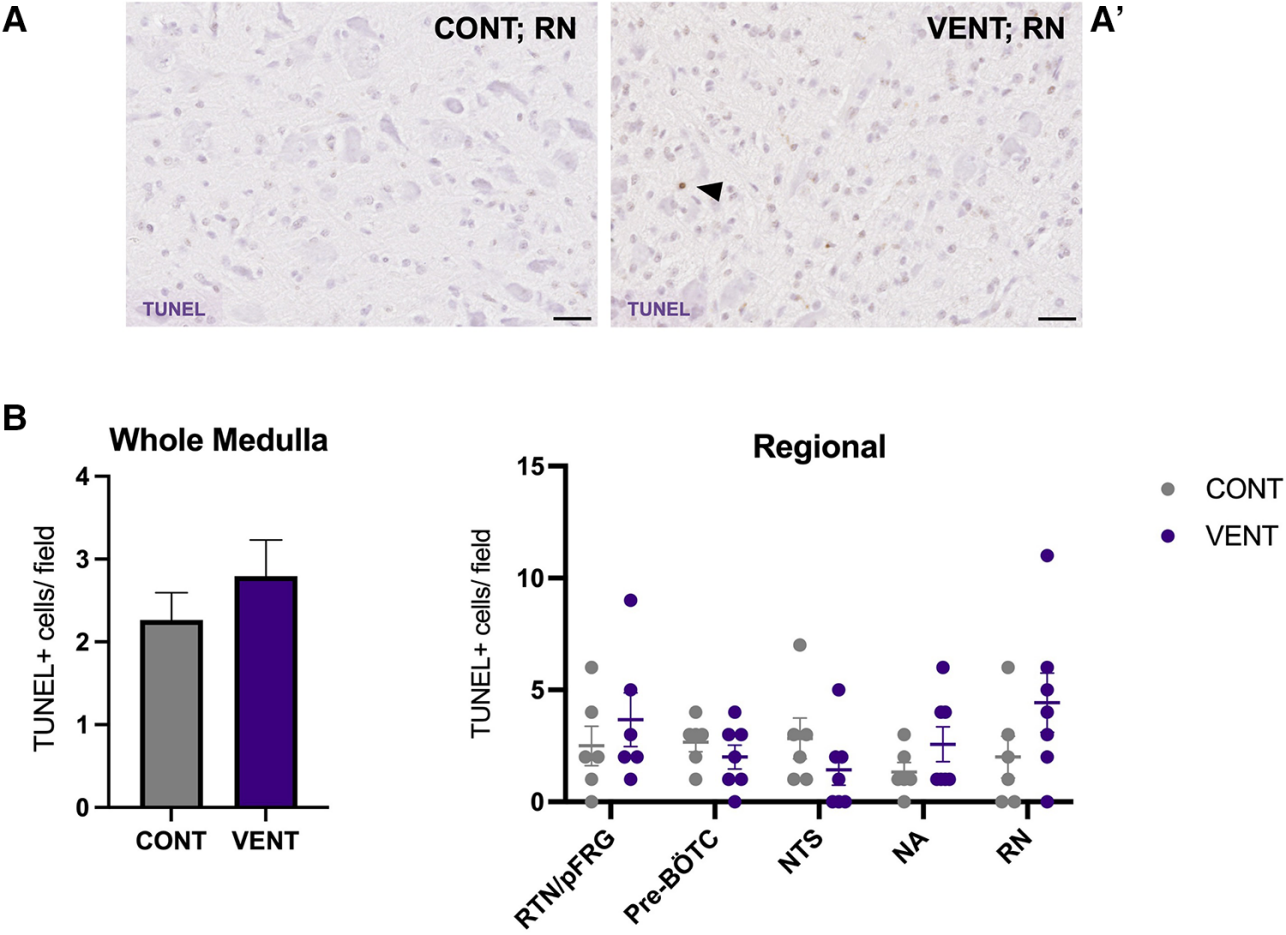
TUNEL-positive cells in brainstem respiratory centres. Representative photomicrographs of TUNEL + staining in the RN of CONT (**A**) and VENT (**A’**) exposed fetuses. Black arrows point to cells with positive staining. (**B**) TUNEL + cells per field in the whole medulla and regional respiratory centres: retrotrapezoid nucleus and parafacial respiratory group (RTN/pFRG), preBötzinger complex (Pre- Bötc), nucleus ambiguus (NA), nucleus tractus solitarius (NTS) and raphe nucleus (RN) in control (CONT; grey circles; *n* = 6) and ventilated (VENT; purple circles; *n* = 7) groups. Data are mean ± SEM.

In the medulla, GFAP + astrocyte staining (measured as % area fraction/field of view) was not significantly higher in the VENT group compared to CONT (*p* = 0.054, Figure 7B). Similarly, the % area fraction of GFAP + stained astrocytes was not significantly higher in the RTN/pFRG (*p* = 0.057) and the pre-BÖTC (*p* = 0.067) in the VENT group compared to CONT. The total number of IBA-1 + cells were increased in the RN (*p* = 0.033, Figure 8B). Subclassification of IBA-1 + cells showed that the number of ramified IBA-1 + microglia were lower in the pre-BÖTC of the VENT group compared to CONT (Figure 8B). The numbers of IBA-1 + ameboid microglia were higher in VENT group compared to CONT in the medulla (*p* = 0.004), NTS (*p* = 0.002) and the RN (*p* < 0.0001; Figure 8C). In the medulla, numbers of caspase-3 positive cells undergoing karyorrhexis or vacuolisation were not different between CONT and VENT (Figures 9A,B) groups, and there were no differences between groups within individual brainstem regions (Figure 9A’ and 9B’; RTN/pFRG, pre-BÖTC, NA, NTS and RN). There were no differences in numbers of TUNEL positive cells between CONT and VENT groups in the whole medulla or individual brainstem respiratory centres (Figure 10B).

## Discussion

Very preterm infants <32 weeks of gestational age often require extensive periods of respiratory support (5, 6) which increases the risk and severity of preterm brain injury leading to long term neurodevelopmental deficits (35). Many of these infants have difficulty in establishing independent breathing following prolonged periods of respiratory support. Several studies have reported decreased brainstem volumes in preterm infants following prolonged mechanical ventilation (36–38), however, little is known about the effects of prolonged mechanical ventilation on inflammation and injury within brainstem respiratory centres. We investigated whether 24 h of *in utero* mechanical ventilation would increase systemic inflammation and cause inflammation and injury within the brainstem respiratory centres of preterm fetal sheep. We found that mechanical ventilation increased systemic concentrations of cytokines IL-6 and IL-8, cerebrospinal fluid concentrations of IP-10, and increased numbers of ameboid microglia compared to controls but did not cause cell death in brainstem respiratory centres. Taken together, these data indicate that mechanical ventilation induced a systemic inflammatory response that was associated with histological evidence of inflammation within brainstem respiratory centres.

We demonstrated a prolonged increase in systemic proinflammatory cytokines, IL-6 and IL-8, in response to mechanical ventilation. The cause of the systemic inflammatory response during mechanical ventilation is thought to arise from the repeated volutrauma and barotrauma associated with mechanical ventilation of the structurally immature lung (39, 40). In preterm fetal sheep of a similar gestational age, mechanical ventilation for 12 h using similar tidal volumes was associated with pulmonary inflammation and tissue injury (24, 41). These data, together with our findings, indicate that mechanical ventilation can cause a pulmonary inflammatory response which results in a systemic inflammatory cascade, that is initiated within hours, and remains elevated through the first 24 h of mechanical ventilation. This inflammatory response was activated despite achieving a tidal volume (2.8 ml/kg) that was only slightly above the expected dead space volume of the lung. Thus, it is likely that only a small part of the lung was aerated, and that the entirety of the tidal volume was entering and ventilating a small region, resulting in inflammation and injury. Importantly, this highlights that injuring even a small portion of the lung has potentially significant downstream consequences.

Consistent with our findings, an acute systemic inflammatory response during respiratory support was shown in term and late preterm human neonates, who showed increased plasma pro-inflammatory cytokines IL-8 (2.5-fold), IL-1β (7.5-fold) and TNF (10-fold), and decreased anti-inflammatory cytokine IL-10 (by 90%) from only 2 h after the initiation of mechanical ventilation (21). Furthermore, ventilation of extremely preterm infants for 14 days increases circulating concentrations of IL-1β, TNF and IL-8 (22). In our study, we showed IL-6 and IL-8 were increased throughout the 24 h of IUV. Upregulation of IL-6 and IL-8 are strongly associated with adverse neurodevelopmental outcomes (22, 29, 42, 43). For example, increased plasma IL-6 in preterm neonates is an independent risk factor for intraventricular haemorrhage and periventricular leukomalacia during the early postnatal period (44, 45). Increased circulating IL-8 in preterm neonates has been associated with an increased requirement for mechanical ventilation and impaired cognition in early childhood (22, 46).

The mechanisms through which the brainstem may become injured are potentially multifactorial. Systemic inflammation has the potential to cause an immune response in the central nervous system (CNS). There are several possible mechanisms by which this may occur. Firstly, systemic cytokines can stimulate the production of matrix metalloproteases, causing breakdown of the normally impermeable BBB (47, 48). Studies have shown a reduction in tightness and an increase in leakiness of the BBB following inflammation, allowing for the entry of systemic pro-inflammatory cytokines and peripheral leukocytes into the central nervous system (47, 49). Following ventilation at high tidal volumes, the BBB of fetal lambs was more permeable, as shown by increased blood vessel protein extravasation in the white matter (33). Secondly, systemic pro-inflammatory cytokines may enter the CNS via tissues that are devoid of a BBB, including the choroid plexus which is the site of the blood-CSF barrier (49, 50). Like the BBB, the blood-CSF barrier is also vulnerable to systemic inflammation. IL-1β, IL-6 and IFN have been implicated in compromising the integrity of this barrier and allowing for entry and an inflammatory response to occur within the CSF which may further manifest into the brain/brainstem tissue (51, 52). Lastly, systemic inflammatory cytokines can cause an immune response in the CNS by entering the brain and the brainstem via saturable transport. Whilst the role of the BBB is to regulate the movement of a number of proteins and cells into the brain, systemic cytokines are able to enter the CNS via BBB which serves as an interface for cytokine transport and entry (53). This mechanism has been demonstrated in a murine model where radioactively labelled IL-1β and IL-6 were able to cross an intact BBB via saturable transport (54). It is possible that through any of these mechanisms, increased systemic IL-6 and IL-8 may have increased inflammation within the CSF and brainstem tissue of ventilated preterm fetal sheep.

The entry of pro-inflammatory cytokines and peripheral leukocytes into the CNS can lead to microglial and astrocyte recruitment, proliferation, and activation (49, 55, 56). The initiation of a localised inflammatory response within the central nervous system (CNS), including activation of glial cells, can promote chronic CNS inflammation via the release of pro-inflammatory mediators including cytokines, reactive oxygen and nitrogen species, excitatory amino acids, and BBB dysfunction, allowing further infiltration of inflammatory mediators (39, 57, 58). Following 24 h of IUV, the numbers of ameboid microglia were higher in the NTS of ventilated fetal sheep when compared to controls. The NTS is composed of a compact network of neurons and is the first site of cardiovascular afferent terminals including carotid body afferents responsible for coordinating cardiorespiratory responses to hypoxia (59, 60). Vagal afferents innervating the airways and the lungs terminate in the NTS, responsible for coordinating immune to brain communication (61, 62). A previous study highlighted a similar increase in the proportion of activated microglia and IL-1β within the NTS following systemic inflammation induced by intravenous infusion of LPS to 8-week old male Wistar rats (63). The NTS expresses receptors for PGE_2_ (64) Activation of these receptors is associated with a reduction in firing amplitude and frequency in respiration-related brainstem neurons and consequently breathing activity (65). The NTS is the first site of termination and integration of respiratory sensory information, responding to pulmonary stretch receptors (66). Therefore, it is postulated that inflammation in the NTS may present clinically as an inhibition to respond and adapt to respiratory-related challenges including, but not limited to, hypoxia and hypercapnia. By contrast, microglial activation within the NTS has also been associated with increased cardiorespiratory reflex sensitivity. These data raise the possibility that microglial activation within the NTS may promote an endogenous protective response to systemic inflammation (60, 61).

The number of ameboid microglia within the RN of ventilated fetal sheep were higher compared to unventilated fetal sheep. Neurons of the RN are highly sensitive to changes in systemic pH and CO_2_. In response to acidosis, chemosensitive neurons of the RN release neurotransmitters such as serotonin (5-HT), which is thought to regulate increased ventilation and modulate autonomic control in response to changes in blood pH (67). In acute brainstem slices, neurons of the RN increase their action potential firing rate 3-fold in response to mild changes in pH (pH of 7.4 to 7.2) (68). The RN has been previously shown to be vulnerable to the effects of systemic inflammation (69–71). The RN is located adjacent to the cerebral aqueduct, a major source of CSF flow. Previous studies have shown that LPS administration to brainstem slices reduce the number of serotonergic neurons in the RN (72). Systemic administration of IL-1β, TNF and LPS have also been shown to alter serotonergic neurotransmitter excitability and release of 5-HT, as well as microglial activation in the RN (70, 73, 74). It is possible that inflammation within the CSF of ventilated fetal sheep may account for the increased activation of microglia within the RN. The RN is responsible for modulating responses to central chemosensitivity and respond to several visceral afferents (75). Together with the NTS, both respiratory centres have a central role in modulating responses to altered partial pressures of oxygen and carbon dioxide. Studies have increasingly highlighted that systemic transfusion of LPS, IL-1β and PGE_2_ result in apnoea and autoresuscitation inhibition following anoxia (11, 16, 76). PGE_2_ has also been shown to inhibit fetal breathing movements in several large animal models (14, 77). It is therefore postulated that increased inflammation in brainstem respiratory centres, particularly those responsible for controlling peripheral and central chemosensitivity, may result in inadequate responses to hypoxia, inhibition of gasping as well as limited capacity to auto resuscitate. This may clinically present as an increased requirement for respiratory support, as well as increased desaturations, apnoeas and bradycardias as described previously (12). Further studies are now required to determine whether the increased microglial activation within the NTS and the RN impact cardiorespiratory reflex responses and adaptations to chemosensitive changes during and after mechanical ventilation. Furthermore, whilst there is growing evidence that brainstem inflammation/injury may have profound influences on cardiorespiratory control in preterm infants, its influence on immediate and long-term neonatal outcomes, particularly neurological, have not been elucidated, and warrants further investigation.

Mechanical ventilation did not significantly increase astrocyte coverage in the medulla of VENT fetuses compared to CONT. Nevertheless, there was a trend for increased astrocyte coverage within the medulla (*p* = 0.054). Similarly, we observed a trend for increased astrocyte coverage within the RTN/pFRG and the pre-Bötc of VENT fetuses compared to CONT (*p* = 0.057 and 0.067 respectively) after 24 h of mechanical ventilation. Astrocytes within the brainstem respiratory centres are highly chemosensitive and respond to physiological changes in pH by calcium fluctuations and release of adenosine triphosphate (ATP) (78). The pre-Bötc and the RTN/pFRG are medullary respiratory centres (8). The pre-Bötc is responsible for generating inspiratory rhythmogenesis and plays a role in controlling upper airway patency to regulate the passage of air into the lungs (8, 79). The RTN/pFRG contains neurons that provide rhythmogenic expiratory activity and is an important site of chemoreception (80). Furthermore, the astrocytes of the RTN/pFRG are highly chemosensitive, responding to decreases in blood and brain pH by increasing intracellular calcium and release of ATP which in turn activates chemoreceptor neurons to increase breathing (78). Pre-Bötc astrocytes function in a similar way, where they have important signalling function which are mediated by vesicular release of gliotransmitters that control breathing rate and rhythm, and regulate respiratory responses to hypoxia and hypercapnia (81). For example, blocking vesicular release in Pre-Bötc astrocytes reduced resting breathing rate, decreased breathing rhythm variability, impaired respiratory responses to hypoxia and hypercapnia, and reduced exercise capacity in adult male rats. These data demonstrate Pre-Bötc astrocytes play a key role in adaptive respiratory responses during conditions of increased metabolic demand (81), which may include perinatal infection/inflammation (82). We did not observe changes in arterial blood gases or lactate concentrations in the VENT group compared to CONT. These data are consistent with previous studies (24) and confirm that *in utero* ventilation does not alter placental function or affect the fetal cardiopulmonary circulation, where the lungs do not support gas exchange and receive minimal (10%–15%) right-ventricular output (83). Overall, our data suggests that astrogliosis and microgliosis in key brainstem respiratory centres may alter their function. Whether this inhibits or augments the ability to breath independently or adapt to physiological challenges in the preterm fetus or neonate is an area that requires further investigation.

We observed no changes in brainstem mRNA levels of pro-inflammatory cytokines, markers of tissue injury and death, and prostaglandin synthesis in VENT fetuses compared to CONT after 24 h of IUV. It is possible that differences in gene expression profiles did not manifest within the 24-hour IUV period. The moderate level of brainstem gliosis and the lack of cell death and apoptosis observed in the VENT group compared to CONT would support this hypothesis. Alternatively, it is possible that differences in mRNA expression between the groups had resolved by the time of tissue collection. We have previously shown increased mRNA expression of pro-inflammatory cytokines IL-1β, IL-6 and IL-8 in the white matter of preterm lambs ventilated with higher tidal volumes (7–15 ml/kg) within 2 h of ventilation onset (84, 85). However, these differences had resolved after 24 h (86).

The experimental model of *in utero* ventilation has several advantages, including ventilation of preterm sheep at gestations younger than what would be feasible *ex utero*, and in the absence of other factors required for supporting preterm neonates that in of themselves can cause brain inflammation and injury (24, 25). For example, the intact placental circulation maintains cardiovascular stability and supports nutritional, and oxygen demands of the fetus (87–89). Overall, this allows us to improve our understanding of the impact of mechanical ventilation on the preterm brainstem in isolation of additional factors required for supporting mechanically ventilated preterm neonates. However, the use of the ovine model of prematurity does come with limitations. The timing of development of the respiratory system and the central nervous system of the fetal sheep differs. At 112 d gestation, the fetal sheep lung is equivalent to human lung development between 22 and 24 weeks, defining the extremely preterm infant (27). However, white matter development equates to approximately 30–35 weeks’ gestation, the very or moderately preterm infant (27, 28).

## Conclusion

This study has described for the first time that moderate inflammation is present within the brainstem respiratory centres of preterm fetal sheep following 24 h of *in utero* mechanical ventilation. We demonstrated that ventilation results in a sustained increase in systemic pro-inflammatory cytokines over 24 h. Furthermore, we show moderate changes to microglial morphology towards an activated state within the NTS, and a trend for increased astrocyte coverage in ventilated fetuses, particularly within the RTN/pFRG and the pre-Bötc. Given that many preterm neonates are ventilated for significantly longer durations than 24 h (90), further studies are required to determine the impact of longer durations of mechanical ventilation on brainstem inflammation and injury. In addition, determining whether these histological changes result in functional consequences to cardiorespiratory control and independent breathing in preterm neonates requires further study.

## Data Availability

The original contributions presented in the study are included in the article/Supplementary Materials, further inquiries can be directed to the corresponding authors.
